# Assisting species differentiation and taxonomic classification by hyperspectral imaging: an example from the parasitic plant realm

**DOI:** 10.1186/s13007-025-01498-y

**Published:** 2026-01-11

**Authors:** Vasili A. Balios, Samuel Ortega, Karsten Heia, Anna Avetisyan, Kirsten Krause

**Affiliations:** 1 Department of Arctic and Marine Biology, UiT The Arctic University of Norway, Tromsø, Norway; 2 Department of Seafood Industry from the Norwegian Institute of Food, Fisheries and Aquaculture Research, Nofima AS, Tromsø, Norway; 3 Scientific Center of Agrobiotechnology, Armenian National Agrarian University, Yerevan, Armenia

**Keywords:** *Cuscuta*, Hyperspectral images, Host/parasite differentiation, Species classification

## Abstract

**Background:**

*Cuscuta*, a genus of parasitic plants, poses a threat to global agriculture by infesting a wide variety of economically important crops and facilitating the transmission of plant viruses. Accurate species identification is crucial for management but is traditionally based on morphological traits that require expert knowledge, limiting accessibility and early detection. Hyperspectral imaging, a technique that captures detailed reflectance information across hundreds of narrow and contiguous wavelength bands, offers the potential to non-invasively monitor plant health with high precision. This study aimed to explore whether hyperspectral imaging, combined with machine learning algorithms, can accurately differentiate between host plant tissue and parasitic *Cuscuta* species and further distinguish among different species within the genus.

**Results:**

Hyperspectral images were collected in both the visible-near infrared and short-wave infrared ranges, followed by preprocessing and segmentation of plant material from the background. The Normalized Difference Vegetation Index method yielded the most consistent segmentation performance. Random Forest and Neural Network models trained on segmented pixels achieved high classification accuracy and balanced F1 scores of approximately 0.97 in both binary (host versus parasite) and multiclass (species-level) classification. Feature selection using a genetic algorithm and an iterative elbow method successfully reduced the number of spectral bands needed for accurate predictions, identifying key wavelengths associated with chlorophyll content and other biochemical markers.

**Conclusions:**

This study demonstrates the effectiveness of hyperspectral imaging combined with machine learning for identifying and classifying parasitic *Cuscuta* species. The findings highlight the potential of this approach for rapid, non-destructive field diagnostics and precision agriculture applications. As imaging hardware continues to improve and become more affordable, such integrated systems could be deployed in real-world crop monitoring and management to mitigate the impact of parasitic plants on global food production.

**Supplementary Information:**

The online version contains supplementary material available at 10.1186/s13007-025-01498-y.

## Introduction

*Cuscuta* species, commonly known as dodder, are a genus of parasitic plants that infect a wide range of hosts. Many of these hosts are globally important crops such as legumes, sugar beet, tomato, onion, garlic, carrot, oilseeds, fodder crops, coffee, tea, and ornamental plant species [1–3]. The infection is facilitated through haustorial connections that establish a direct link to the host’s vascular system, creating a strong nutrient and water sink, which significantly reduces crop yield [2, 4]. *Cuscuta* can also facilitate the transmission of various viruses throughout crops which can further affect crop yields and health negatively [5, 6]. Although a substantial amount of research on *Cuscuta* has been aimed at preventing and managing infections, there are reports of *Cuscuta* species moving or widening their distribution range with a changing climate [3, 7, 8]. However, since the species determination is difficult, relying heavily on experts who use mainly distinctive flower characteristics that are too advanced for a layperson, there can be elements of uncertainty connected to reported sightings.

*Cuscuta* species have already established broad geographical distributions across tropical, subtropical, and temperate regions, attributed to their broad host range and adaptability to diverse habitats. However, their current distribution is expected to expand further due to climate change. Climatic shifts, such as increased temperatures and altered precipitation patterns, may significantly enlarge suitable habitats for several *Cuscuta* species, potentially allowing these parasites to invade new regions. Recent studies using predictive distribution models have shown that areas suitable for certain *Cuscuta* species could expand dramatically, potentially increasing by up to sixfold by 2070 [7, 8]. Furthermore, habitat specificity of *Cuscuta* species is not merely a function of host availability, but also strongly influenced by abiotic factors such as soil characteristics [9]. This complex interplay between biotic and abiotic factors implies that climate-induced changes in soil conditions may further facilitate or restrict *Cuscuta’s* spread, underscoring the necessity of integrated ecological approaches for monitoring and management strategies under changing environmental conditions [9].

**Fig. 1:**
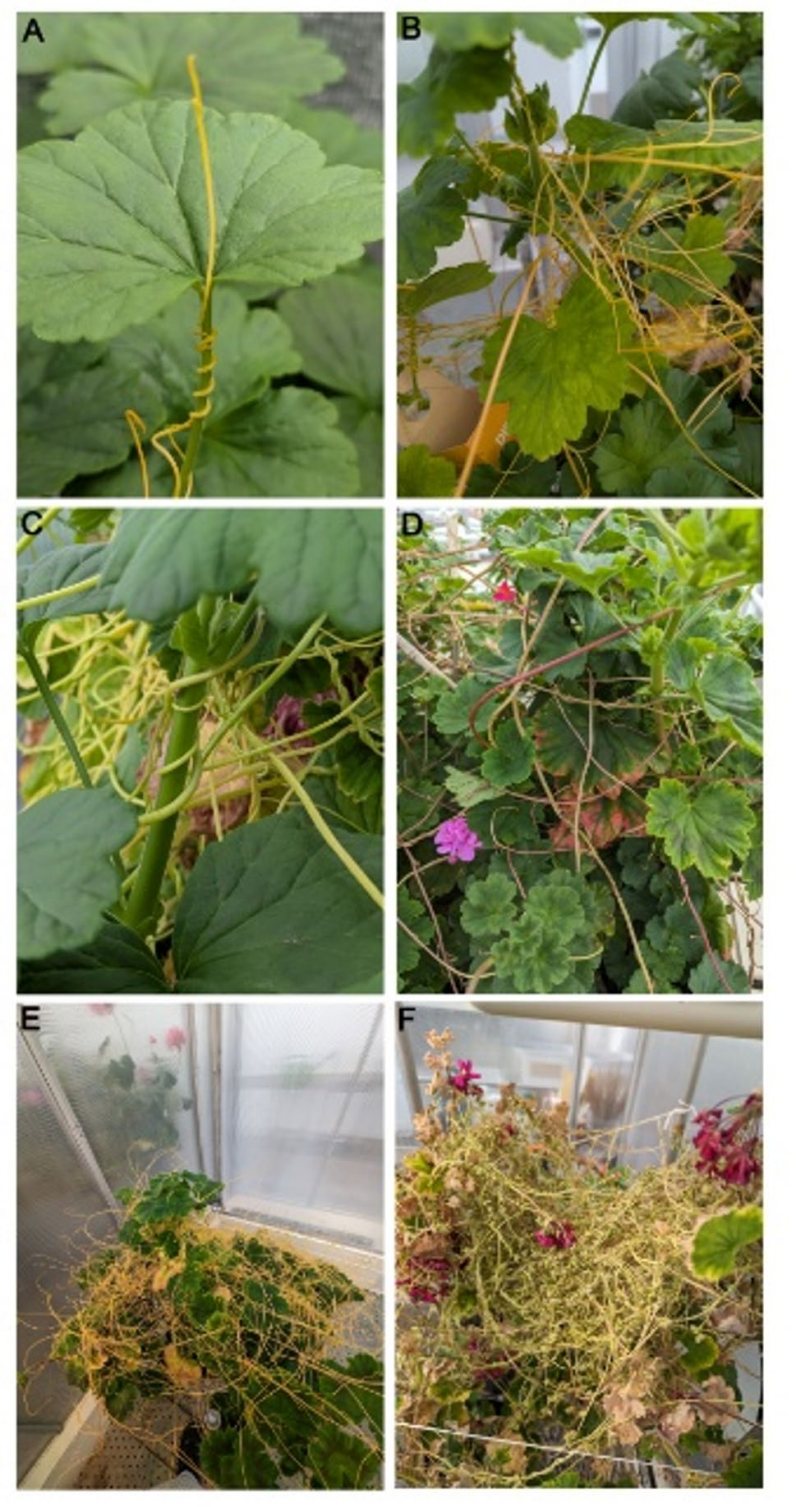
*Cuscuta* species on the same host (*Pelargonium zonale*). **A** *Cuscuta campestris*. **B** *Cuscuta platyloba.* **C** *Cuscuta reflexa.* **D** *Cuscuta monogyna*. **E ** and ** F** Examples of heavily infested* P. zonale*. All plants were grown under the same conditions

*Cuscuta* plants exhibit a characteristic growth habit as they wind and branch around the host plant, closely attaching to stems, leaves, and petioles (Fig. 1). During growth, the parasite forms specialized organs known as haustoria along its stem, particularly on the host-facing side. These haustoria initiate from meristematic tissue within the inner cortex layers of the parasite stem, forming an adhesive disc that firmly attaches to the host’s surface. From the adhesive disc, a specialized intrusive organ, the haustorium proper, penetrates into the host tissues using a combination of mechanical force and enzymatic degradation. Within the host, flexible searching hyphae grow into the vascular tissues, maturing into feeding hyphae once they establish contact with host xylem and phloem vessels, facilitating direct nutrient transfer [10–12].

Resistance to *Cuscuta* in crops is rare [13], though tolerant [14] or resistant genotypes exist [15–17]. Management includes intercropping with non-host plants [1, 18], mechanical removal [2, 19, 20], and chemical treatments that often risk host damage [21, 22]. Certain effective herbicides are heavily regulated [23]. Emerging approaches, such as manipulating light conditions [24] or biological controls [1, 25, 26], require further validation. Combining multiple methods tailored to specific host-parasite systems is likely necessary [27]. The development of a rapid, reliable field warning system for early detection of *Cuscuta* is crucial for sustainable and targeted parasite management.

Hyperspectral imaging (HSI) is an emerging technology gaining popularity due to its ability to measure the interaction of light with matter, allowing for the chemical identification of materials within an imaging modality. The integration of HSI with machine learning and artificial intelligence enables detailed studies across various fields, particularly in agriculture. By capturing spatial and spectral information in the form of a 3D data cube, where each pixel records reflectance across numerous wavelengths, HSI significantly surpasses traditional RGB imaging by detecting subtle variations in chemical composition and allowing early, accurate identification of plant stress, biomass, nitrogen content, and disease states [28–32]. Compared to multispectral methods, HSI provides superior precision in measuring leaf area index, crop biomass, leaf nitrogen concentration, and distinguishing between crop species, facilitating earlier detection of physiological changes linked to plant health [33–36]. Despite these advantages, challenges such as high data volume and noise still exist [37, 38]. For applications in the agricultural and plant ecology fields, HSI is often used in longer range aerial approaches. However, in other fields such as, for example, in the food industry and in medical sciences, there are many reports of the use of these cameras in closer range applications [39–41]. In phytopathology, HSI is particularly valuable for its ability to detect disease-specific spectral signatures associated with plant biochemical compounds, such as chlorophyll, water, and nitrogen, across defined wavelength ranges [42–45].

The plant genus *Cuscuta* exhibits differences in chlorophyll and other pigment levels, especially compared to autotrophic plants, but also between the different subgenera, and even between some of its species [46]. Species of the subgenus *Grammica* are often yellow/orange due to high levels of carotenoids while in the subgenus *Monogynella* chlorophylls may (e.g. in *C. reflexa*) or may not (e.g. in *C. monogyna*) be dominating while carotenoids do not accumulate to visible levels (Fig. 1). Exploiting these differences in HSI-based approaches would widen the powerful potential of HSI in AI-supported pathogen detection and plant species identification in both field and controlled experimental contexts. Standard RGB image assessment can in theory be automated to distinguish host and *Cuscuta* (depending on the greenness of the host stems), and may potentially be suitable to distinguish some *Cuscuta* species with a more distinct appearance. However, the yellow shoots of the large species group belonging to the subgenus *Grammica* (e.g. *Cuscuta campestris* and *Cuscuta platyloba* (Fig. 1A, B)) are indistinguishable in RGB imaging. HSI is not restricted to visible earmarks but can take advantage of other metabolic differences and use them as taxonomic markers rather than relying only on more subtle pigmentation variations. While multiple studies have already described the HSI-mediated detection of parasitic plants from the Orobanchaceae family and also from Mistletoe [47–50], *Cuscuta* has so far remained uncharted terrain with respect to the potential of HSI. The only current publication investigating hyperspectral imaging in combination with *Cuscuta* focusses on the effects of *Cuscuta* infection on the host plant, not on the parasite [51]. Another paper has demonstrated the use of drones/unmanned aerial vehicles for *Cuscuta* infestation detection, however without incorporating HSI [52]. Thus, there is a large need to expand the use of this powerful technology to the parasite *Cuscuta*.

The primary objective of this research is to examine the feasibility of distinguishing between the host and the parasite through hyperspectral image analysis. Furthermore, the study aims to determine whether it is possible to use the specific spectral characteristics of different *Cuscuta* species for species determination. To achieve these goals, different machine learning algorithms were used. In order to interpret these machine learning models, we also investigated which specific bands are relevant for differentiating between the host and the parasites, as well as between different parasite species, using different feature selection algorithms.

## Materials and methods

### Plant material

**Fig. 2:**
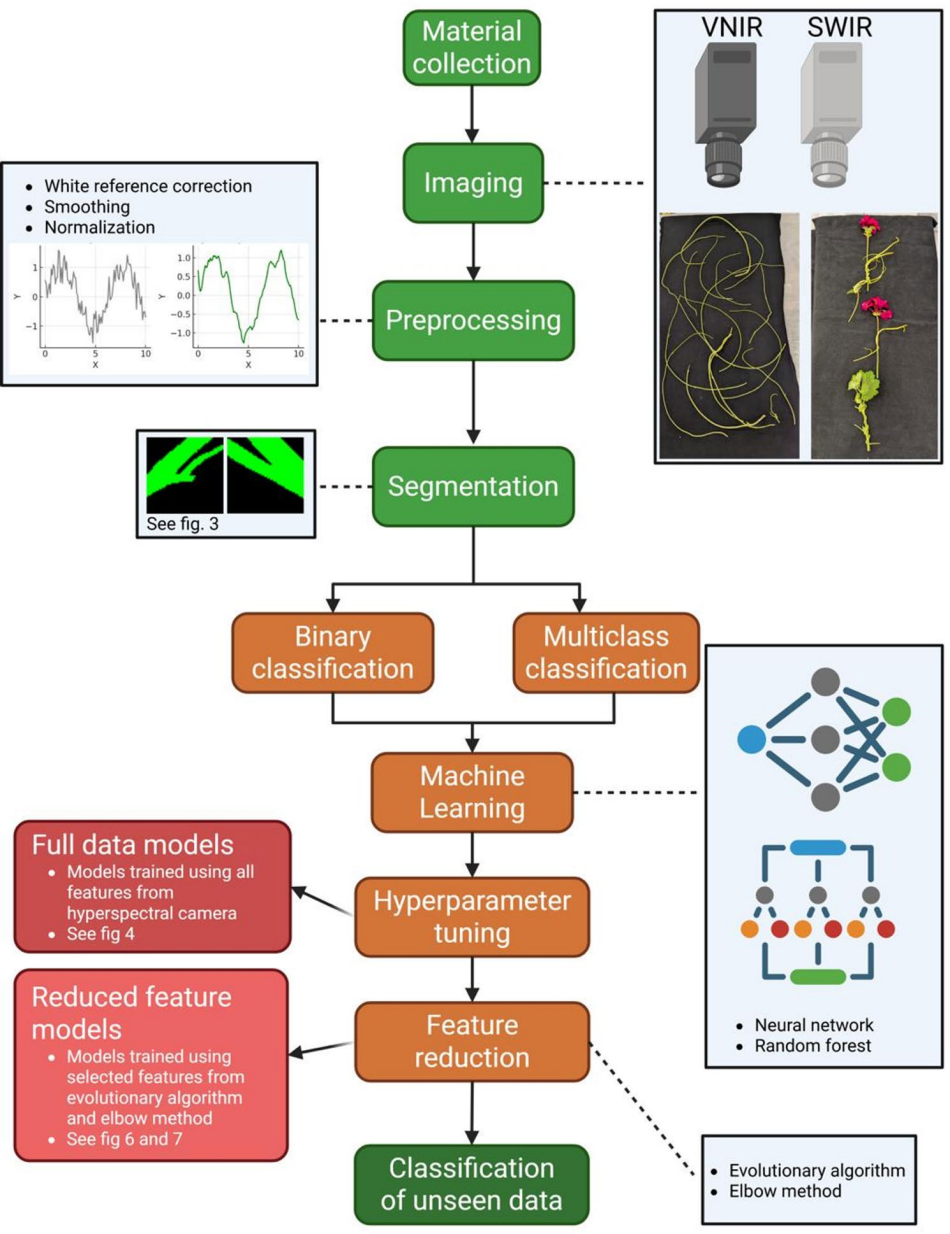
Flow diagram. The diagram depicts each step in the analysis workflow starting with the harvesting of the plant material and their imaging. Preprocessing steps consist of flat-field calibration with the white reference, Savitzky-Golay smoothing and normalization. Segmentation removes background pixels while also labelling the plant pixels with their respective class ("Host", "parasite" (only for binary applications), "*campestris*", "*reflexa*", "*monogyna*"* and *"*platyloba*"). Machine learning algorithms (Random Forests, Neural Network) allows to automate pixel classification in new datasets, requiring hyperparameter tuning and feature reduction to evaluate and unlock their full potential under optimized computational investment

Three *Cuscuta* species that were used in this study (*C. campestris* and *C. platyloba* from the subgenus *Grammica*, and *C. reflexa* from the subgenus *Monogynella*) were obtained from the Botanical Garden of the Christian Albrechts University of Kiel in 2006, while the fourth species, *Cuscuta monogyna*, was collected in Armenia in 2019. All of them have been clonally propagated on *Pelargonium zonale* as a host at the Climate laboratory of UiT The Arctic University of Norway in Holt, Tromsø by placing *Cuscuta* cuttings of approximately 20 cm with the cut end in water and winding them loosely around stems of a new host. The growth conditions for the harvested plant material were as described previously [53] with one exception: The natural daylight was supplemented with artificial LED light (Cosmorrow IR and Cosmorrow grow light LEDs, Gartnerbutikken) (instead of the earlier used fluorescent light tubes) to produce an average light intensity of 80 µmol photons•m-2•s-1 and a light quality with a high far-red component supporting infections. Samples taken for hyperspectral imaging were harvested from well-established feeding *Cuscuta* cultures and each included (i) the infection sites where the parasite twines around and penetrates the *P. zonale* host, as well as free shoot segments, (ii) shoots from the parasite and (iii) non-parasitized stem segments from the host (Fig. 2, inset). Plants were harvested in the morning at irregular intervals over the period of half a year (June to December 2024) before imaging. The following number of shoots were collected and imaged for each species, *Cuscuta reflexa*: 93, *Cuscuta monogyna*: 92, *Cuscuta campestris*: 185, *Cuscuta platyloba*: 92, *Pelargonium zonale*: 40.

### Image acquisition

Hyperspectral imaging was done at the Norwegian Food Research Institute (NOFIMA) in Tromsø. Briefly, in this study, two hyperspectral cameras were used to capture images within the visual and near-infrared (VNIR) and short-wave infrared (SWIR) spectral regions. For the VNIR imaging, a HySpex VNIR-1800 camera (Norsk Elektro Optikk AS, Oslo, Norway) was utilized, providing a spectral coverage from 407 to 995 nm. This camera offers a spectral resolution of 5.5 nm across 186 bands and has 1800 spatial pixels. For imaging in the SWIR region, a HySpex SWIR-384 camera (Norsk Elektro Optikk AS, Oslo, Norway) was used, covering wavelengths between 950 and 2518 nm. Similar to the VNIR device, it features a spectral resolution of 5.5 nm but includes 288 spectral bands and 384 spatial pixels. Both cameras provide a field of view of approximately 300 mm at a working distance of 1000 mm, resulting in pixel sizes of 0.16 mm for the VNIR system and 0.17 mm for the SWIR system. The samples were placed on Styrofoam boards covered with a black cloth for image acquisition.

Maintaining consistent lighting and sample positioning was crucial to ensure data quality across images. The cameras were fixed on a stable mount, and samples were transported beneath them using a conveyor belt moving at a precisely calibrated speed of 102 mm/s. Image capture rates were determined by the cameras’ exposure times, set at 4.8 ms for the VNIR camera and 5.9 ms for the SWIR camera, resulting in effective pixel dimensions of 0.16 × 0.49 mm and 0.17 × 0.6 mm, respectively (Ortega, Lutfi [54]). Each camera system was illuminated using a custom-made Teflon illumination box equipped with 14 internal halogen bulbs, each rated at 50 W (Halogen display/ optic lamp, Osram, Germany). The illumination box featured a narrow slit at the top aligned with the camera’s field of view and an open bottom, providing uniform and diffuse lighting onto the conveyor belt and ensuring consistent broadband illumination across the imaging area. Specific wavelength bands used for both the VNIR and SWIR hyperspectral cameras are detailed in Supplementary Table 1.

### Pre-processing

The full images produced with the cameras were used to crop out smaller images. This cropping step was important to remove most of the background pixels as well as separating sections of the image which contained only one species. This is due to the nature of the image acquisition, which allows multiple species to be imaged at the same time and produces one large, composite image. Thus, the size of each of these sub images are not a standard size and all subsequent analysis was done using these images separated based on species. All cropping was done in Spectronon (Spectronon Pro, Resonon, Bozeman, MT, USA). All other coding/application of algorithms was performed using Python 3, and all graphs were produced in Python with either matplotlib or seaborn [55, 56]. Flat field calibration corrects spatial and spectral non-uniformities in imaging systems by capturing an image of a uniform white reference. This image serves as a baseline to normalize subsequent measurements, ensuring consistent and accurate reflectance or radiance data across the entire field of view. The flat-field calibration with the white reference (Spectralon^®^ Reflectance Material, Labsphere (North Sutton, NH, USA)) was done using the built-in algorithm included in the Spectronon software. All images were then smoothed with the Savitzky-Golay smoothing filter using a window length of 13 and a third-degree polynomial. This is a spectral filter that processes each pixel’s spectrum along the spectral axis. The data was partitioned into 60% train, 20% test and 20% validation splits taking care that pixels from the same image were not shared across different subsets to avoid data leakage. Since images were acquired using a conveyor belt-based imaging system capable of capturing continuous scans (see image acquisition), these composite images were cropped into smaller “tray” images, which were sometimes further subdivided into “tiles”, depending on their size. Tiles located in close spatial proximity were grouped and assigned to the same data split to preserve local spectral and spatial continuity. Conversely, tiles from more distant regions were allocated to different splits to reduce spatial and spectral leakage across subsets.

The data from the train set was then normalized using the MinMaxScaler from the sklearn package [57]. The scaler was saved to use later for normalizing unseen data for classification of new images. This scaler is specific for our experimental setup and we used this consistently for all images taken in this paper.

### Segmentation

To segment plant material from the background, several clustering algorithms were evaluated. This evaluation was necessary as *Cuscuta* material with reduced or lacking chlorophylls and lack of leaves differed too profoundly to indiscriminately apply approaches reported earlier for plants. K-means clustering was applied with two, three, and four clusters using Euclidean distance [58]. This clustering partitions a dataset into *k* distinct, non-overlapping clusters. Each data point belongs to the cluster with the nearest mean, serving as the cluster’s centroid. The algorithm aims to minimize the within-cluster sum of squares (WCSS), effectively reducing the variance within each cluster [58]. Additionally, X-means clustering was tested with three and four clusters to automatically identify an optimal number of clusters within the given range [59]. The X-means algorithm is similar to K-means in that it partitions a dataset into distinct non-overlapping clusters, where the main difference is that the X-means algorithm determines the most optimal number of classes in a user given range [59]. Learning Vector Quantization (LVQ) was also evaluated using three clusters, employing prototype-based classification [60]. LVQ is an algorithm that utilizes prototype-based classification, where each class is represented by one or more prototype vectors. During training, these prototypes are adjusted to accurately represent their respective classes by moving closer to correctly classified data points and away from misclassified ones, effectively partitioning the input space for classification tasks [60]. The Normalized Difference Vegetation Index (NDVI) is a widely used remote sensing indicator to assess vegetation health, density, and biomass. NDVI leverages the fact that healthy vegetation strongly absorbs visible light (particularly red wavelengths) and strongly reflects near-infrared (NIR) light, thus making this a suitable metric to use for our VNIR data. For the VNIR hyperspectral data, the NDVI was computed. The Normalized Water Difference Index (NWDI) is another remote sensing spectral index, specifically designed to identify and monitor water content in vegetation. NWDI is sensitive to the moisture content of plant tissues and helps detect early stages of drought or water stress in plants. It is computed using SWIR and NIR wavelengths. Thus, we utilised NWDI for SWIR data segmentation. For the SWIR data, segmentation was performed using a combination of the Normalized Difference Water Index (NDWI) and K-means clustering.

### Machine learning models used for classification of different classes of pixels

An initial evaluation of models was performed using 10% of the data (Supplementary Fig. 1) utilizing the data mining software WEKA [61] using the following commonly used machine learning models: Random Forest [62], J48 [61], Multi Objective Evolutionary Fuzzy classifier (MOEFC) [63], and Continuous High-resolution Image Reconstruction using Patch priors (CHIRP) [64]. Support Vector Machine (SVM) was attempted but took considerably longer time and was therefore abandoned. Later, we opted for Random Forest and Neural Network classifiers as the Random Forest performed best in the initial evaluation and produced a decision tree based model to compare to a Neural Network type model [65, 66]. Since the different *Cuscuta* species have quite different shoot diameters, their representation in relation to each other and to their hosts generated an imbalance in class representation (e.g. dominance of *C. reflexa* pixels and a slightly underrepresented amount of host pixels) which could have a potentially negative impact for downstream analyses. Hyperparameter tuning was therefore performed to maximize the F1 score and thereby enhance classification performance. The F1 metric gives equal weight to all classes. This ensures that the models that were trained in these experiments performed well across all classes, not just the dominating ones [67, 68].

### Feature selection methods

Feature selection involved two distinct approaches. Firstly, Local Interpretable Model-Agnostic Explanations (LIME) [69] were used to determine the most important features for both Random Forest and Neural Network models. The top-ranked features identified by LIME were then selected to train simplified models using fewer features, following an elbow method, which iteratively uses the best features to train the model. Secondly, a genetic algorithm was implemented to discover optimal subsets of spectral bands. This algorithm evaluated randomly generated subsets of bands based on their F1 scores [68] from both Neural Network and Random Forest models. The best-performing subsets were iteratively refined through mutation and selection until a suitable stopping condition, either a high enough F1 score or a maximum number of generations, was reached. The F1 score condition was set to the best F1 score achieved when the Random Forest and Neural Network are trained using all the features. The maximum number of generations were set to 50 due to time and computation constraints. The final selected subset of features was then used for subsequent training of both Random Forest and Neural Network models. (Full code can be found on GitHub).

## Results

Images were taken from non-infective shoots of each species and from shoots infecting a host stem, resulting in a total of 13 hyperspectral image sets, each generating approximately 2 to 4 gigabytes of data. Since the analysis of such data volumes requires considerable computing power and time, steps to eliminate all non-informative pixels are paramount before commencing with the data analysis. As a first processing step after common pre-processing techniques (cropping, flat-field calibration, smoothing and normalization), the application of a segmentation algorithm for the separation of background pixels from sample pixels was necessary. The sample pixels were then classified to identify informative spectral traits for each parasite as well as for the host as a task that can be supported by supervised machine learning. To make the analysis as effective as possible, we tested further if a reduction of the total features to only those that contribute most to the classification can accelerate analysis. An overview of the overall analysis pipeline and the different algorithms or processes that were contrasted with each other as described in the course of the following chapters is shown in Fig. 2.

### Normalized difference indices perform best for segmentation of *Cuscuta* plant material from background

**Fig. 3:**
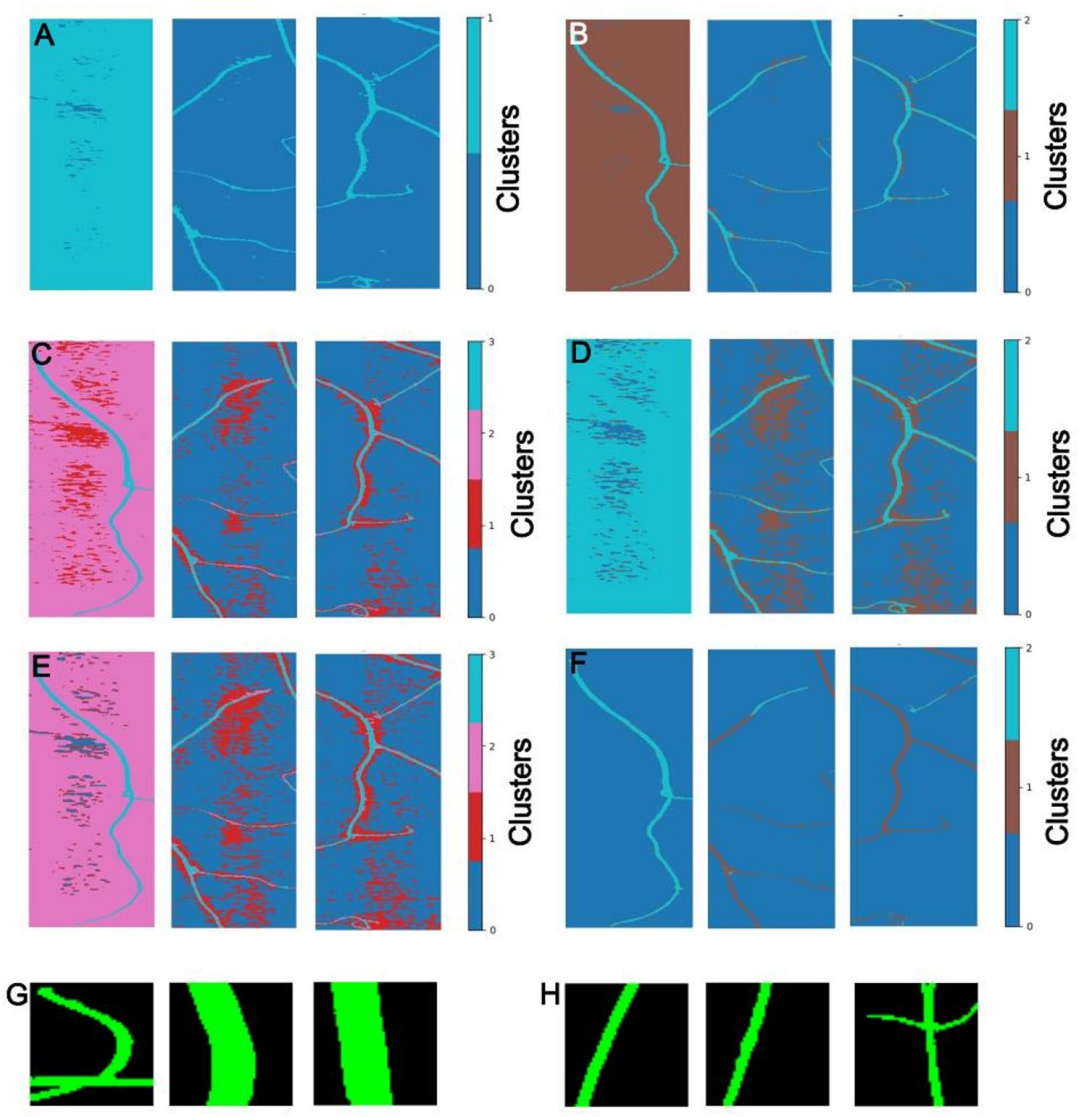
Segmentation of background and plant pixels. The same random subset of images were used to test different algorithms that could be used for segmentation (**A**–**F**). Random subsets were used to test the NDVI and NDWI-kmeans approaches (**G** and **H**), respectively. **A** K-means clustering using two clusters. **B** K-means clustering using three clusters. **C** K-means clustering using four clusters. **D** X-means algorithm using three clusters. **E** X-means algorithm using four clusters. **F** Learning Vector Quantization (LVQ) using two clusters. **G** NDVI based segmentation with a threshold of 0.75, using bands 121 (827.09 nm, NIR) and 64 (620.29 nm, red), specifically used for VNIR data. **H** NDWI with K-means clustering based segmentation, using bands 27 (1098.07 nm) and 136 (1655.20 nm), specifically used for SWIR data

Segmentation was necessary to distinguish background, host (*P. zonale*), and parasite (*Cuscuta* species) pixels within each hyperspectral image cube. To find the most effective way, multiple segmentation methods were evaluated. The application of K-means clustering on the *Cuscuta* and combined *Cuscuta*/host pixel using two clusters provided moderate segmentation quality but lacked consistency across images (Fig. 3A). Increasing K to three clusters consistently improved the separation of plant pixels from background (Fig. 3B). Four clusters highlighted additional variation in background pixels without further enhancing segmentation accuracy (Fig. 3C). X-means clustering was less effective with three clusters, producing significant overlap between plant and background pixels (Fig. 3D). Four clusters with X-means provided better segmentation, clearly differentiating plant material from the background and revealing background variation (Fig. 3E). The K-means and X-means algorithms did not produce consistent results for all plant pixels, and some plant segments had a large amount of background pixels which were clustered with the plant pixels despite our attempts to optimize the number of clusters to segment the plant pixels consistently from the background pixels (Fig. 3A–E). LVQ performed well in segmenting background and plant pixels, revealing more variation within the plant pixels rather than the background (Fig. 3F). However, its computational time was considerably higher compared to other methods.

Utilizing an NDVI-based segmentation improved the separation of plant from non-plant pixels significantly. An NDVI threshold of 0.75, using bands 121 (827.09 nm, NIR) and 64 (620.29 nm, red), was most effective (Fig. 3G). However, for the SWIR data, it was necessary to combine NDWI with K-means clustering using bands 27 (1098.07 nm) and 136 (1655.20 nm) to achieve satisfactory segmentation accuracy (Fig. 3H). NDVI (used for VNIR data) and NDWI-Kmeans (used for SWIR data) used very little time to compute a large amount of data. These algorithms also resulted in only two classes: plant pixels and background pixels. In accordance with the ultimate goal to distinguish between *Cuscuta* species by accurate multiclass classification, the plant pixels (overall 2,224,867 pixels) were then labelled after NDVI based segmentation of VNIR data as “Host” (240611 pixels), *“C. campestris”* (452461 pixels), “*C. reflexa”* (594847 pixels), “*C. monogyna”* (460568 pixels), or “*C. platyloba”* (476380 pixels), respectively. The larger number of *Cuscuta* pixels ensured sufficient representation and robustness of the species classification model of this genus but caused a class imbalance, with pixels for each parasite species outnumbering host pixels by approximately 2:1. To mitigate the effects of this class imbalance, model optimization prioritized the F1 score, representing the harmonic mean of precision and recall, rather than overall accuracy, allowing a more reliable measure of model performance on imbalanced datasets.

### Hyperparameter tuning of random forest and neural network achieves high F1 and accuracy for both binary classification and multiclass classification

The Neural Network model underwent hyperparameter tuning, involving terms related to the number of units, dropout rates, and learning rate. For the Random Forest model, hyperparameters including the number of estimators, maximum depth, maximum features, minimum samples per leaf, and bootstrap method were tuned. A final model was then trained using the best-performing hyperparameters identified during tuning. (Table 1 for summary statistics of models).

The test F1 score and the test accuracy were calculated for each model. Binary models refer to the Neural Network and Random Forest models trained to classify host and parasite tissue while the multiclass models also differentiate between the different *Cuscuta* species. GA = Genetic algorithm, EL = elbow method.

**Table 1:** Summary statistics of each model

Model	Test accuracy	Test F1 score
Binary neural network	0.91	0.95
Binary random forest	0.97	0.91
Multiclass neural network	0.90	0.89
Multiclass random forest	0.84	0.85
GA Multiclass neural network	0.93	0.93
EL multiclass neural network	0.91	0.91
GA multiclass random forest	0.84	0.83
EL multiclass random forest	0.84	0.82

**Fig. 4:**
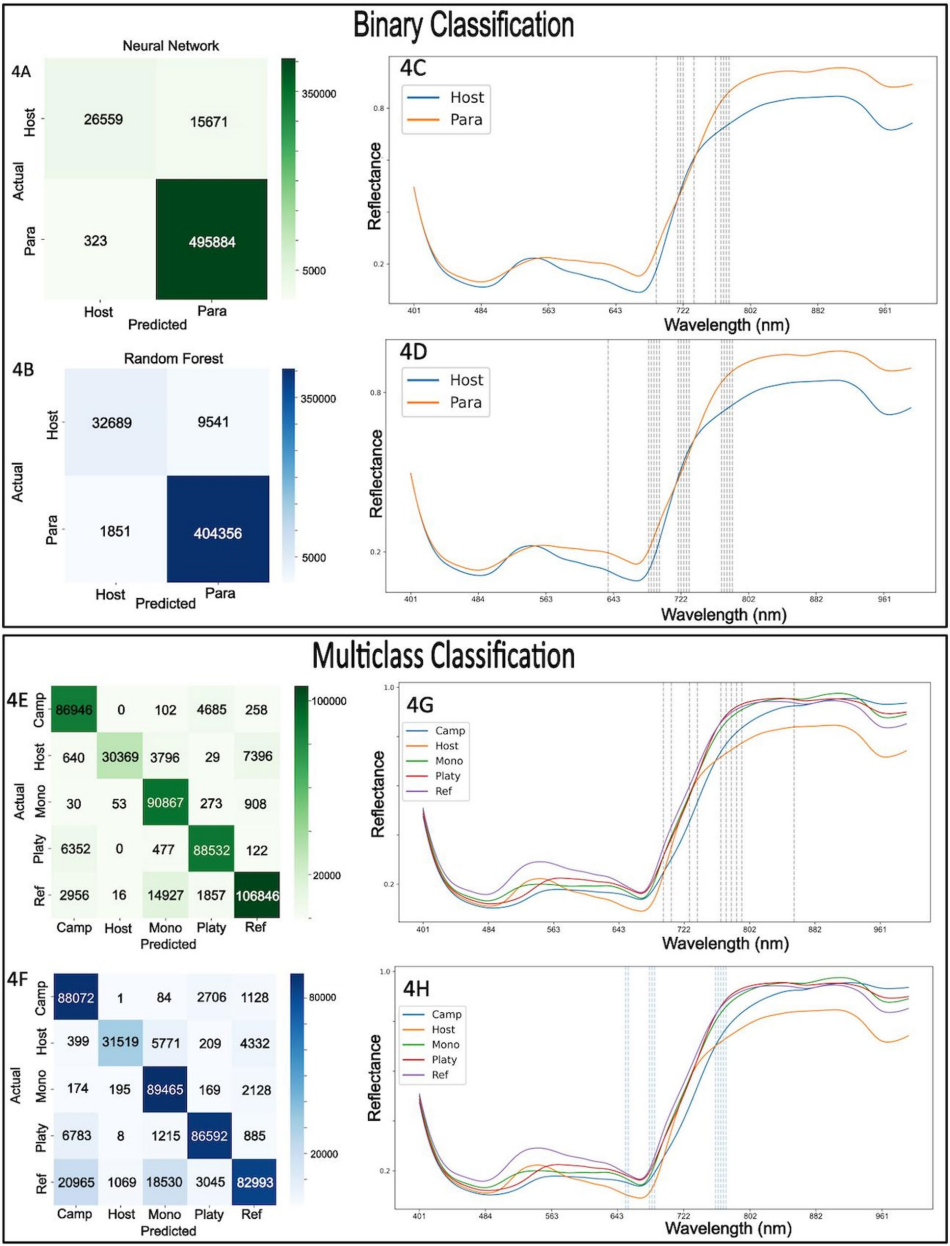
Evaluation of different binary and multiclass classification models. **A** Confusion matrix for Neural Network and **B** Random Forest for binary classification. Pixels predicted as host or parasite compared to the actual label of each pixel. **C**, **D** Mean reflectance for host and parasites with important features for Neural Network (**C**) and Random Forest models (**D**). **E** Confusion matrix for Neural Network and (**F**) Random Forest for multiclass classification. Pixels predicted as host or parasite species (*campestris*,* reflexa*,* monogyna*,* platyloba*) compared to the actual label of each pixel, assigned “semi-supervised” after the segmentation of pixels. **G**, **H** Mean reflectance for host and parasites with important features for Neural Network (**G**) and Random Forest models (**H**). The top ten features for each model (dotted lines) are plotted in relation to the wavelength in **C**, **D**, **G** and **H**

To distinguish host from parasitic pixels, binary classification was first tested. Optimal Neural Network parameters included 384 and 128 neurons in the first and second hidden layers, respectively, with dropout rates of 0.0 and 0.4, and a learning rate of approximately 1.97 × 10^− 4^. Optimal Random Forest parameters were 313 trees, maximum depth of 50, square-root feature selection per split, minimum leaf size of 1, and no bootstrap sampling (Table 1 for summary statistics of models) based on an initial weakly constrained hyperparameter search. Retraining the RF model with shallower maximum depth revealed that both the F1 score and the accuracy levelled off at a maximum depth of approximately 20–25, with no appreciable gains beyond this range, though for the Random Forest models shown in this study the higher initial value of 50 was used. High accuracy was observed in binary classification using both Random Forest and Neural Network models (Fig. 4A, B). Feature importance analysis using LIME identified crucial spectral bands for both models (Fig. 4C, D), highlighting their significance in distinguishing between classes. From the feature importance results it seems that the Random Forest model relies on (finds very important) a cluster of bands between approximately 640 nm and 780 nm for differentiation between host pixels and parasitic pixels (Fig. 4D), which is similar to what is seen for the Neural Network (Fig. 4C), see Supplementary Table 2 for all feature importances. The average spectrum of host and parasitic pixels are significantly different and generally do not overlap.

We used multiclass classification to distinguish between all the *Cuscuta* species as well as host plants. Optimal Neural Network parameters included 384 and 256 neurons in the first and second hidden layers, respectively, with dropout rates of 0.1 and 0.4, and a learning rate of approximately 2.1 × 10^-4^. Optimal Random Forest parameters were 176 trees, maximum depth of 29, square-root feature selection per split, minimum leaf size of 1, and no bootstrap sampling. The multiclass classification accuracy for the four *Cuscuta* species was generally high (Fig. 4E, F), but better for the Neural Network compared to the Random Forest (Table 1), although some confusion was noted between *C. campestris* and *C. platyloba* of the subgenus *Grammica*, as well as *C. reflexa* and *C. monogyna* of the subgenus *Monogynella*, indicating that there are more distinct features separating the two subgenera than the species within them.

Misclassification was generally observed for host pixels that were occasionally misinterpreted as belonging to *Cuscuta* with more confusion being observed with *C. reflexa* and *C.monogyna* than with the other two species. Analysis of spectral band importance further clarified bands critical for species differentiation (Supplementary Table 2 for importance of all features). These important bands seemed to be associated with chlorophyll reflectance, this was visually confirmed through spectral analysis (Fig. 4G, H). The SWIR data worked marginally well for binary classification allowing us, in most cases to differentiate between host tissue and parasitic tissue. However, the SWIR data classification accuracy was notably lower for multiclass classification compared to VNIR data, rarely allowing to distinguish between the different *Cuscuta* species that was mostly being classified as *C. monogyna* (Supplementary Fig. 2). Subsequent analyses thus focused primarily on VNIR data due to its superior accuracy.

**Fig. 5:**
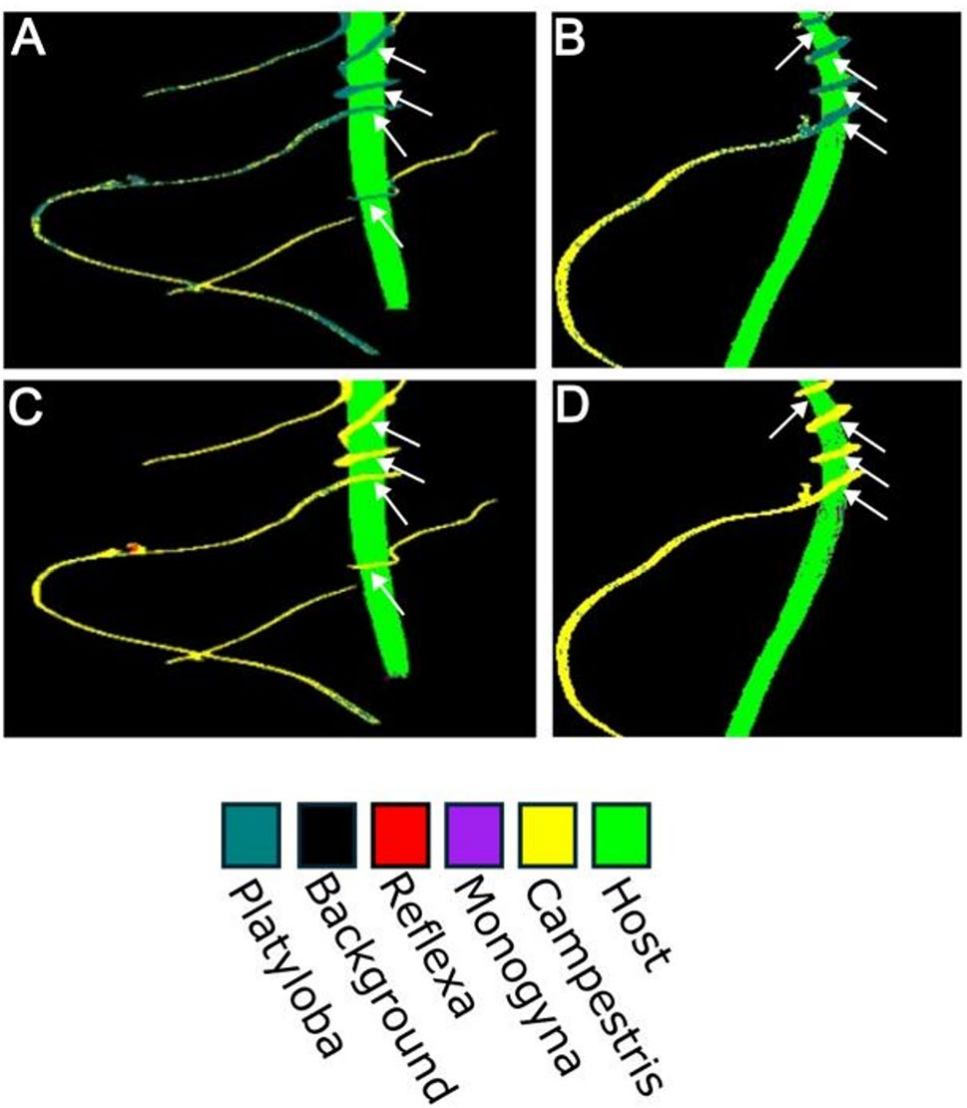
*Cuscuta campestris* classification with potential of subclass classification. **A** and **B** Neural Network trained without pixels from infection sites. Infection sites (marked by white arrows) show tendencies for misclassification. **C** and **D** Same images but classified with a Neural Network which was trained with pixels taken from active infection sites on the host. Infection sites (marked by white arrows) are no longer extensively misclassified

Higher misclassification rates were noted for pixels at parasite-host interaction sites, indicating distinct spectral characteristics at these locations (Fig. 5). Retraining the multiclass Neural Network with pixels specifically from active infection sites significantly reduced misclassification, improving the accuracy of the analysis when images with more complex information are subjected to HSI classification. This may also suggest that parasitic pixels at active infection sites may have a shifted spectrum compared to parasitic tissue further from an infection site, which is a finding whose significance and dynamics are still unknown and which would warrant more attention in the future. Overall, however, we could demonstrate that it is possible to produce models that are able to differentiate between host and parasite and, more importantly, between different species of *Cuscuta*. Ultimately, among the two models, the Neural Network was preferred for its compact file size (2 MB) compared to the Random Forest model (1.72 GB), a reduction by a factor of close to 850. This advantage further supports the use of Neural Networks for large-scale or real-time applications. The Neural Network also performs better than the Random Forest model (Table 1).

### Genetic algorithm and elbow method achieve high F1 score and accuracy using less features

**Fig. 6:**
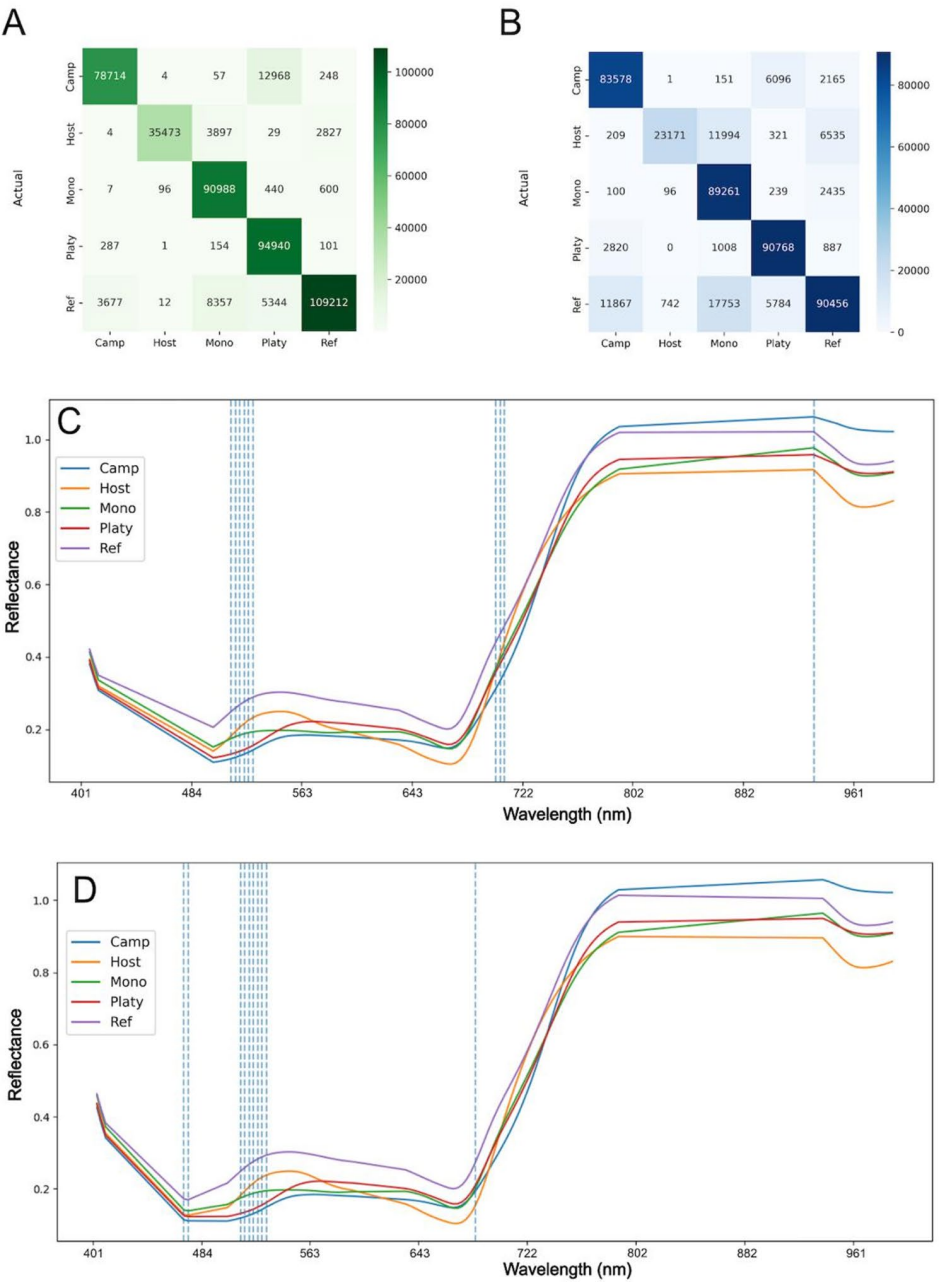
Elbow Method application to Random Forest and Neural Network models. **A** Confusion matrix of the Neural Network and **B** Random Forest after applying the elbow. Feature importances were sorted from most important to least important and was calculated using LIME. **C**, **D** Mean reflectance for host and parasites with important features for Neural Network (**C**) and Random Forest models (**D**). The top ten features for each model (dotted lines) are plotted in relation to the wavelength

**Fig. 7:**
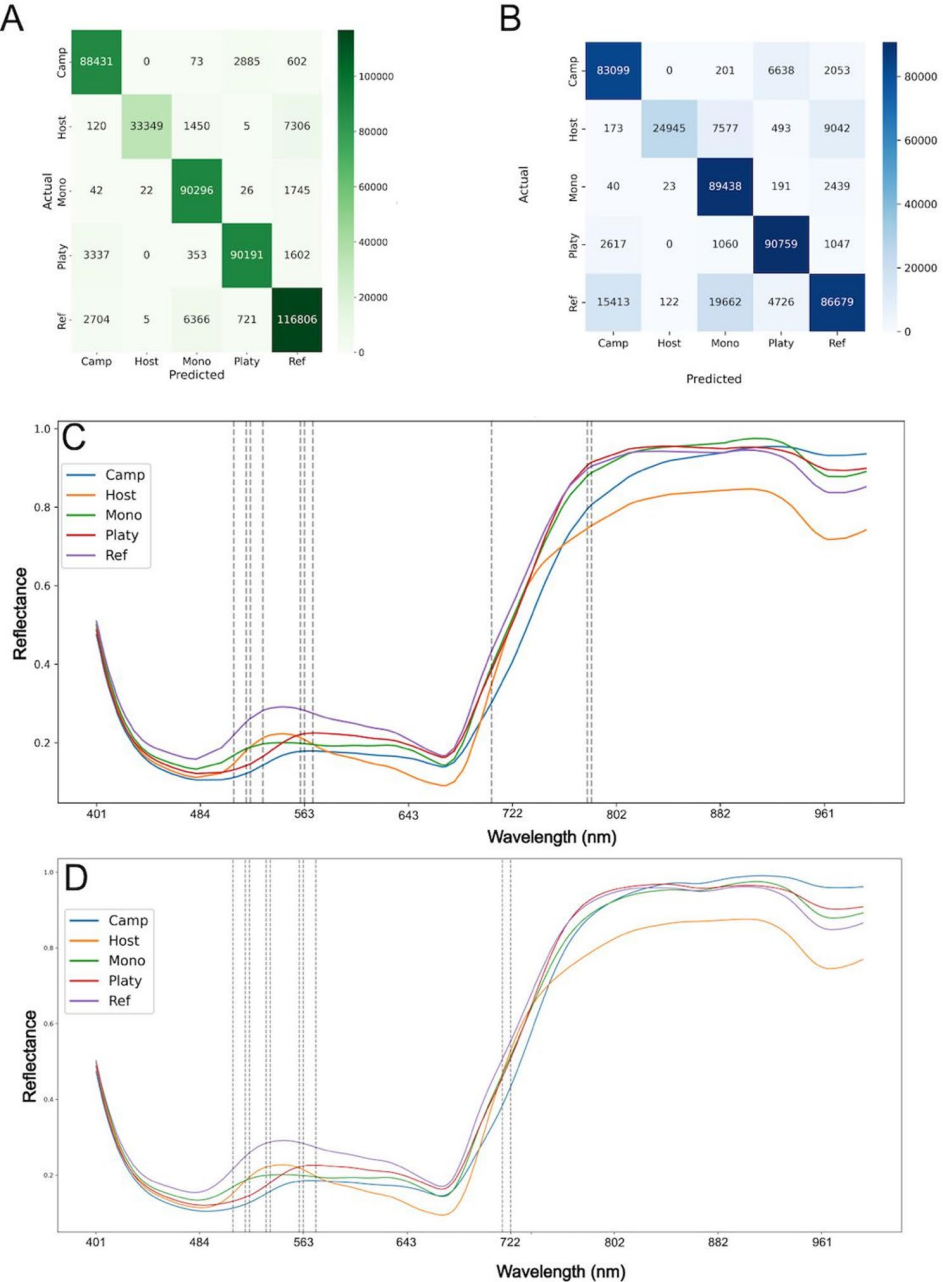
Genetic Algorithm application to Random Forest and Neural Network models. **A** Confusion matrix of the Neural Network and **B** Random Forest after applying the genetic algorithm. **C**, **D** Mean reflectance for host and parasites with important features for Neural Network (**C**) and Random Forest models (**D**). The top ten features for each model (dotted lines) are plotted in relation to the wavelength. Feature importances were sorted from most important to least important and was calculated using LIME

Feature reduction was evaluated comparatively using Genetic Algorithm and Elbow Methods to identify the minimal number of spectral bands needed for accurate classification (Figs. 6 and 7). The feature selection was conducted on the validation set and then applied to the test set. The best multiclass classification models produced in the previous analysis were then used for testing both algorithms. After applying the Elbow Method or the Genetic Algorithm, respectively, these models for multiclass classification using either Random Forest or Neural Networks produced similar F1 scores compared to the “full feature” models (Figs. 6 and 7).

The majority of classes were correctly classified as can be seen in the confusion matrices for both the Elbow Method (Fig. 6A, B) as well as for the Genetic Algorithm (Fig. 7A, B). For the multiclass classification, we were able to reduce the number of features while maintaining F1 scores and accuracy. The greatest reduction was seen using the Genetic Algorithm in combination with the Neural Network, where less than half (92 out of 186) of the total features were needed to obtain a comparable result. The Elbow Method (Fig. 6C, D) was able to reduce the number of features as well but not to the same degree as the Genetic Algorithm which performed better overall (Fig. 7C, D). In conclusion, it is possible to use less data and get comparable accuracy in less computing time, compared to when all the features are being used. This demonstrates the feasibility of simplifying datasets while retaining high classification accuracy for *Cuscuta* species. Through this selection we have also identified the minimal important combination of features which are required to differentiate host pixels from *Cuscuta* pixels as well as differentiating between different *Cuscuta* species.

**Fig. 8:**
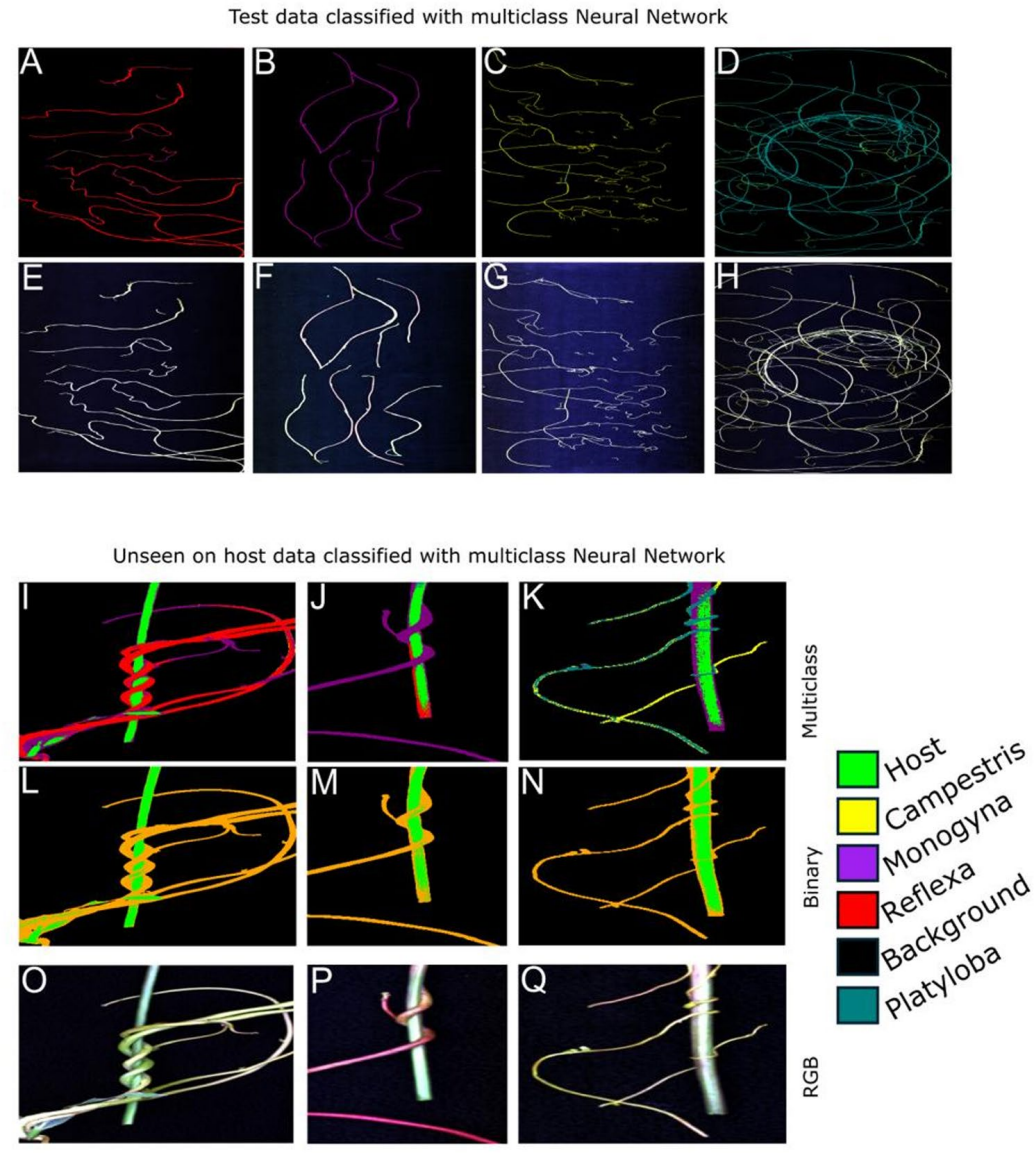
Example of classification using the final Neural Network model. **A**–**D** Classification map of *C. reflexa*, *C. monogyna*, *C. campestris* and *C. platyloba* respectively, after each pixel is classified with the final multiclass Neural Network model. These images were part of the test set, unseen during model training, but imaged similarly to all other training and validation data. **E**–**H** Falsely colored RGB images *C. reflexa*, *C. monogyna*, *C. campestris* and *C. platyloba* respectively. **I**–**K** Classification map of *C. reflexa*, *C. monogyna*, *C. campestris* respectively, after each pixel is classified with the final multiclass Neural Network model. **L**–**N** Classification map of *C. reflexa*, *C. monogyna*, *C. campestris* respectively, after each pixel is classified with the final binary Neural Network model. These images were completely unseen and more complex than the images used during model training. **O**–**Q** Falsely colored RGB images of each species respectively. Each pixel is colored according to the model prediction. The color-coding key for the individual classes is given on the lower right

## Discussion

Species distinction in the genus *Cuscuta* has long been challenging due to their morphological similarities and complex taxonomy, making accurate identification largely dependent on specialist knowledge. Furthermore, tracking changes in *Cuscuta* distribution over time traditionally requires intensive field surveys and expertise in plant systematics. Novel methodologies leveraging widely available technologies, such as imaging combined with artificial intelligence, present a promising solution, enabling even non-specialists to identify *Cuscuta* species, potentially at least to the subgenus level, using specialized HSI cameras once adequate pipelines and reference databases are established. HSI has emerged as a powerful tool in phytopathology for disease detection, species differentiation, and the assessment of plant health both under controlled conditions and in field applications [70, 71]. The current study evaluates the effectiveness of HSI combined with machine learning techniques in distinguishing various *Cuscuta* species from host plants, aiming to overcome existing bottlenecks related to identification accuracy, consistency, and practical applicability, and providing a first step towards the establishment of faster AI-supported field diagnostics for parasitic plants.

Initially, for segmentation of plant from background pixels, we explored several approaches (Fig. 3). However, these methods showed inconsistent results across different image datasets. Transitioning to the NDVI significantly improved segmentation consistency and processing speed for images taken with the HySpex VNIR-1800 camera (407 to 995 nm). NDVI is commonly utilized in multispectral and hyperspectral imaging contexts, particularly for identifying plant material and assessing plant health [72, 73]. We determined that an NDVI threshold value of 0.75 effectively distinguished plant pixels from background across all species tested (*C. campestris*, *C. platyloba*, *C. monogyna*, *C. reflexa*, and *Pelargonium zonale*) (Fig. 3G, H). For the SWIR data produced by the HySpex SWIR-384 camera (950 to 2518 nm), in contrast, NDWI combined with K-means was necessary for an appropriate level of segmentation between plant and background. However, we found that the SWIR data was inferior for distinction between species than data from the VNIR range. This may be due to what the SWIR data is capturing in our plants. SWIR data is better at detecting water content, cellulose, lignin, phenolics, senescence, and some macro nutrients [74–76]. Whereas it seems that the defining characteristics such as carotenoids and chlorophyll content which are important for classifying the *Cuscuta* species, are captured with the VNIR data. The SWIR data was not able to distinguish different *Cuscuta* species (Supplementary Fig. 2. Species classification). Binary classification performed better but still not as good as the VNIR data (Supplementary Fig. 2. Binary classification). However, it is worth noting that it should be investigated whether combining both data can improve classification of species and binary classification.

We trained two distinct models, a Neural Network and a Random Forest, for both binary classification (host vs. parasite) and multi-class classification (host and individual *Cuscuta* species). Both models achieved comparable accuracy and F1 scores (Table 1). However, the Neural Network performed slightly better. Notably, species grouped in the same subgenus (*C. campestris* and *C. platyloba* in subgenus *Grammica*; *C. reflexa* and *C. monogyna* in subgenus *Monogynella*) were more frequently misclassified as each other [77] (Fig. 4) than the species belonging to different subgenera. The frequent confusion between *C. campestris* and *C. platyloba* aligns with visual observations, as these species are difficult to differentiate based on their appearance. Similarly, the misclassification between *C. reflexa* and *C. monogyna* likely results from their evolutionary proximity, which could reflect similarities in biochemical composition and spectral signatures despite differences in their appearance based on their pigment composition.

Feature importance analysis revealed distinct spectral bands critical for classification. For both models, bands clustered between 640 and 800 nm emerged as highly significant (Fig. 4). These bands correspond primarily to chlorophyll-related reflectance, aligning with observed differences in chlorophyll content between parasitic *Cuscuta* species and autotrophic hosts [46]. In this wavelength zone, known as the Red Edge (~ 680 to 750 nm), light absorption changes from strong absorption by chlorophyll a around 662 nm (red light) to strong reflectance of far-red and near infra-red wavelengths. Such wavelengths have previously demonstrated predictive accuracy for chlorophyll and carotenoid contents [78, 79]. Wavelengths within the range of ~ 400–850 nm typically relate to chlorophyll content, while ~ 970–1250 nm, 1450 nm, and 1950 nm bands correlate strongly with water content, and 400–515 nm, 570 nm, and 720 nm are indicators of nitrogen content [43–45]. Such spectral markers can substantially enhance species differentiation capabilities. The significantly lower chlorophyll content in *Cuscuta* species compared to autotrophic hosts, alongside species-specific carotenoid content variations, provide critical spectral signatures, potentially enabling precise identification and monitoring of parasitic infestations. These differences effect the spectrum of the plant, for example, at the red edge which may be utilised by the models for classification, and we do see that there are multiple bands at the red edge which are important for classification. However, it will be important to probe whether these spectral “fingerprints” are subject to dynamic changes under different environmental conditions, in different stages of their life cycle or on different hosts before such methods can be fully exploited. To improve classification of more complex data, where the close association of host and parasite entails a large potential for mixed pixels, methods which involve fuzzy classification (such as Fuzzy Possibilistic C-Means and Modified Possibilistic C-Means) may help to resolve mixed pixels [80] and should be considered. These approaches may also help resolve miss-classified pixels which are seen in Fig. 5A, B as well as Fig. 8I**–**N.

We further utilized a Genetic Algorithm to optimize the number of spectral bands needed for accurate classification. This reduced the necessary bands from 186 to approximately 80 while retaining high classification accuracy for both models (Fig. 6). Genetic Algorithm approaches have previously been successfully applied to feature selection in image classification tasks [81, 82]. With our datasets the Genetic Algorithm computing outperformed the Elbow Method that could not compete in terms of efficiency and robustness. An explanation may lie in the way how the genetic algorithm randomly utilizes different combinations of features, thus retaining potentially hidden interactions between different features, while the elbow method simply iteratively uses the most important features which may result in leaving out these hidden interactions between features.

*Pelargonium zonale* is a commonly used host for *Cuscuta* species, and in this study, it served primarily as a proof of principle to allow a direct comparison among several parasite species for which currently no published hyperspectral data exist. Future research should extend this approach by incorporating additional host plants to verify the robustness of the identified signature bands across different contexts. Follow-up studies, thus should supplement controlled greenhouse experiments with field studies to validate and expand on our findings for specific crops, investigating amongst others how early *Cuscuta* infections on a plant or in a field can be detected and how other parameters can influence the performance of the models.

Although this study is primarily a proof of concept, it demonstrates that HSI coupled with machine learning can differentiate *Cuscuta* species from host plants and from one another based on their abundant shoot tissue. That the model performance declines somewhat in areas where *Cuscuta*-entwined host are present (Fig. 8) suggests that the model may be able to detect physiological differences occurring at the very infection sites, which is of academic interest, but it does not confine its utility for indicating parasite presence as such, or for discrimination groups of species as long as sufficient shoot material is present in the images. Previous studies employing HSI for weed detection provide parallels and support for these conclusions [71, 83]. We believe that our data give reasons to cautious optimism that HSI will in the future also serve as a supporting tool in the field of taxonomy and may assist species discrimination where tell-tale features (e.g. from flowers) are not available.

## Conclusion and remarks

HSI has already been demonstrated to have substantial utility in phytopathology, particularly in detecting and quantifying plant biochemical components. In our study we showed that this technology can not only be used for the identification of an infection (binary classification of parasite vs. host) but moreover also to differentiate between groups of *Cuscuta* species. We found that for this purpose the VNIR data worked well while the SWIR data was only capable of binary classification. Future work should integrate both these data and determine if there is an increase in accuracy for both binary and species classification. We highlight how integration of HSI and machine learning may be utilized for addressing key challenges in taxonomy and field monitoring thus possibly permitting to detect *Cuscuta* outbreaks earlier and to follow distribution patterns more easily. Zhou et al. [84] have shown that special lenses are capable of expanding the spectral information gatherable with standard RGB cameras into the hyperspectral range. HSI cameras which are mounted on tractors or smaller unmanned vehicles (rather than longer-ranged mounted cameras such as UAVs) would be able to monitor fields for early *Cuscuta* outbreaks, so that action can be taken before a full-blown infestation can occur. However, such applications will likely require re-calibration of the models to perform well in field conditions setting the scope of future tasks.

If species level information on *Cuscuta* can be derived from field acquired HSI data, a feasibility supported by our results, this would markedly enhance the quantification and modeling of their spatiotemporal distribution dynamics in response to temperature and other environmental drivers. This capability would improve forecasts of *Cuscuta* emergence and spread, enabling farmers to implement proactive management measures before the parasite has manifested itself. As HSI technology will become cheaper and more accessible in the future these approaches have great potential to be implemented in the field assisting farmers and scientists in the future.

## Supplementary Information

Supplementary material 1. Table 1. Wavelengths of bands. The exact wavelength of each band is reported for the VNIR and SWIR cameras.

Supplementary material 2. Table 2. Feature importances of models. The feature importances (from most to least important) are reported for the binary and multiclass models for both Random Forest and Neural Network. The feature importances were calculated using LIME.

Supplementary material 3. Fig. 1. Different classification algorithms compared. A comparison of commonly used algorithms on a small subset of data to determine the best to go forward with. Random Forest, J48 – tree based, Multi Objective Evolutionary Fuzzy classifier (MOEFC), Continuous High-resolution Image Reconstruction using Patch priors (CHIRP). Each model was trained and evaluated 3 times to get the average.

Supplementary material 4. Fig. 2. Classification of pixels with Random Forest and Neural Network models using SWIR data. Top panel shows the binary classification of each species followed by the species-specific classification using Random Forest models. The bottom panel shows the binary classification of each species followed by the species-specific classification using Neural Network models.

## Data Availability

The scripts for data analysis are available from https://github.com/VasiliBalios/HySpecML.
