# Supplementary figures and images for: Assisting species differentiation and taxonomic classification by hyperspectral imaging: an example from the parasitic plant realm
Source: Plant Methods. 2026 Jan 11;22:14. doi: 10.1186/s13007-025-01498-y (PMC12882463; doi:10.1186/s13007-025-01498-y)

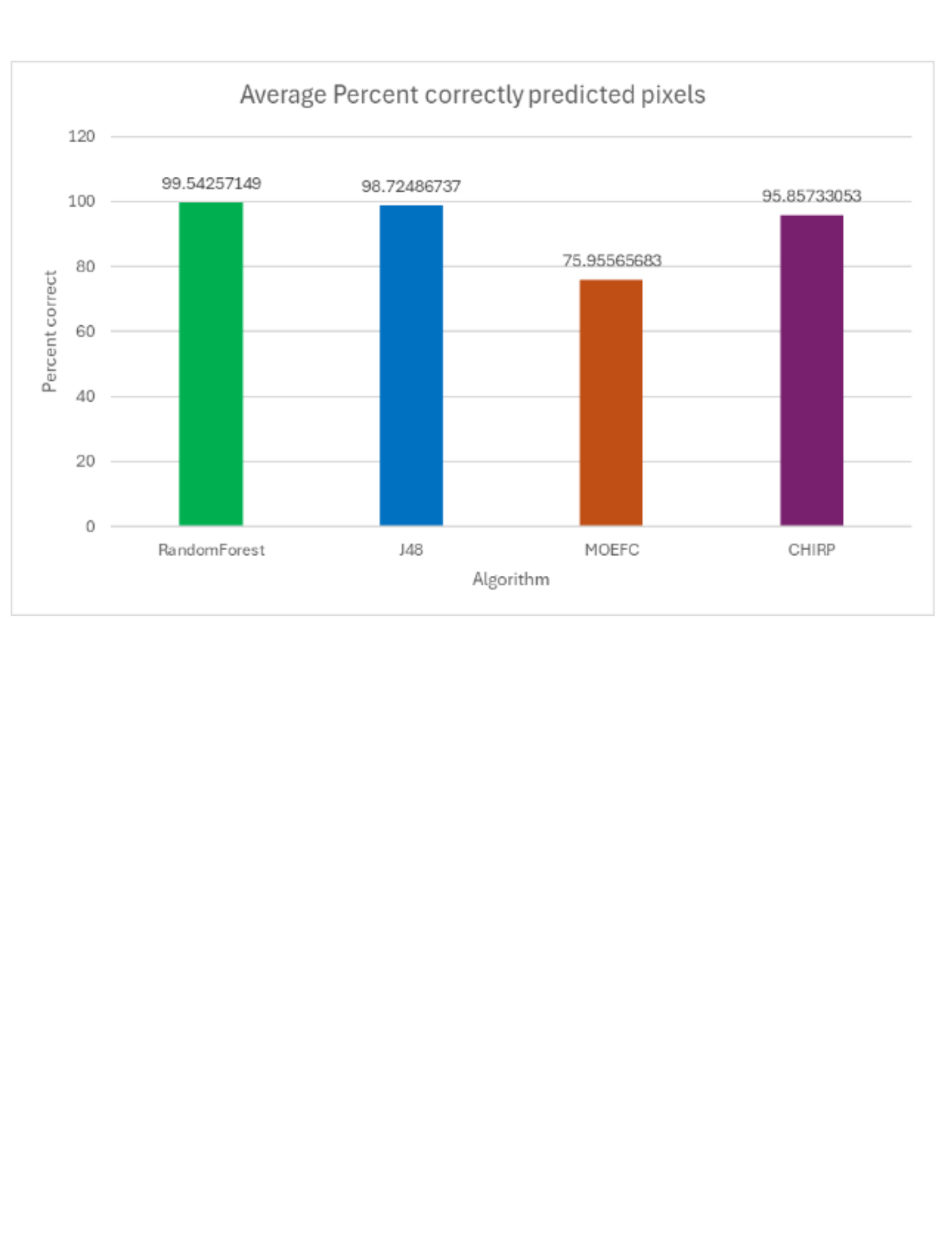

Supplement: Supplementary file 3 — Supplementary material 3. Fig. 1. Different classification algorithms compared. A comparison of commonly used algorithms on a small subset of data to determine the best to go forward with. Random Forest, J48 – tree based, Multi Objective Evolutionary Fuzzy classifier (MOEFC), Continuous High-resolution Image Reconstruction using Patch priors (CHIRP). Each model was trained and evaluated 3 times to get the average. [file 13007_2025_1498_MOESM3_ESM.tiff]

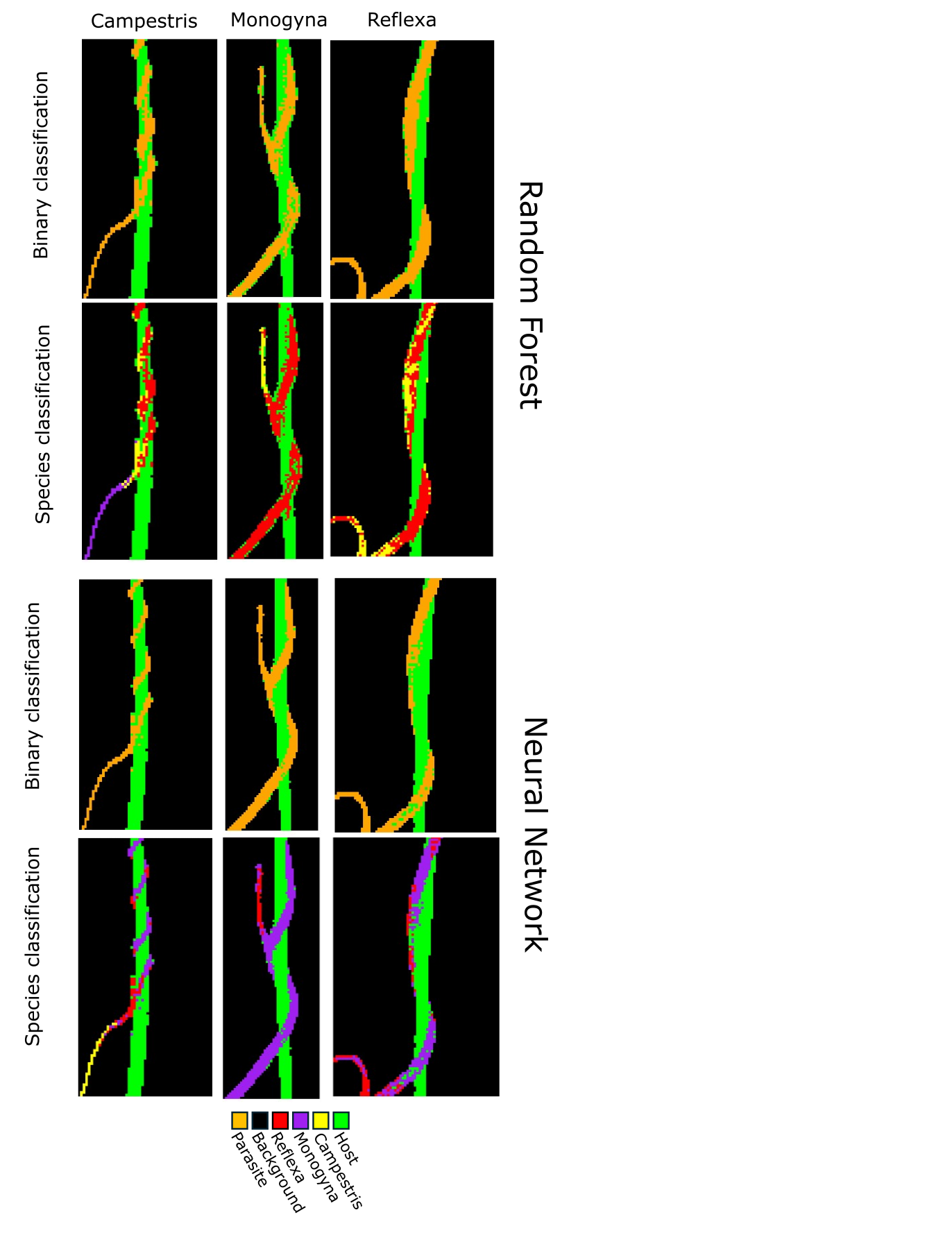

Supplement: Supplementary file 4 — Supplementary material 4. Fig. 2. Classification of pixels with Random Forest and Neural Network models using SWIR data. Top panel shows the binary classification of each species followed by the species-specific classification using Random Forest models. The bottom panel shows the binary classification of each species followed by the species-specific classification using Neural Network models. [file 13007_2025_1498_MOESM4_ESM.tiff]
